# Testing survey-based methods for rapid monitoring of child mortality, with implications for summary birth history data

**DOI:** 10.1371/journal.pone.0176366

**Published:** 2017-04-25

**Authors:** Eoghan Brady, Kenneth Hill

**Affiliations:** Johns Hopkins School of Public Health, Baltimore, Maryland, United States of America; University College London, UNITED KINGDOM

## Abstract

**Introduction:**

Under-five mortality estimates are increasingly used in low and middle income countries to target interventions and measure performance against global development goals. Two new methods to rapidly estimate under-5 mortality based on Summary Birth Histories (SBH) were described in a previous paper and tested with data available. This analysis tests the methods using data appropriate to each method from 5 countries that lack vital registration systems. SBH data are collected across many countries through censuses and surveys, and indirect methods often rely upon their quality to estimate mortality rates.

**Methods and findings:**

The Birth History Imputation method imputes data from a recent Full Birth History (FBH) onto the birth, death and age distribution of the SBH to produce estimates based on the resulting distribution of child mortality. DHS FBHs and MICS SBHs are used for all five countries. In the implementation, 43 of 70 estimates are within 20% of validation estimates (61%). Mean Absolute Relative Error is 17.7.%. 1 of 7 countries produces acceptable estimates. The Cohort Change method considers the differences in births and deaths between repeated Summary Birth Histories at 1 or 2-year intervals to estimate the mortality rate in that period. SBHs are taken from Brazil’s PNAD Surveys 2004–2011 and validated against IGME estimates. 2 of 10 estimates are within 10% of validation estimates. Mean absolute relative error is greater than 100%.

**Conclusions:**

Appropriate testing of these new methods demonstrates that they do not produce sufficiently good estimates based on the data available. We conclude this is due to the poor quality of most SBH data included in the study. This has wider implications for the next round of censuses and future household surveys across many low- and middle- income countries.

## Introduction

Child mortality rates have been among the most important indicators used to monitor development among low- and middle-income countries (LMICs) through the Millennium Development Goals (MDGs) and other global development initiatives. Between 1990 and 2015 the Under-5 Mortality Rate (U5MR) is estimated to have declined by more than half [1]. Child mortality data are also of value for designing and assessing the impact of health interventions. Child mortality estimates for LMICs, most of which lack the accurate civil registration systems that are the conventional source of child mortality data, are primarily derived from household surveys which collect Full Birth Histories (FBH) from women of reproductive age. FBHs consist of dates of birth and if relevant ages at death for each live-born child reported by the women surveyed. Such surveys are typically carried out at intervals of 5 years or more due to the extensive questionnaires and interviewer training required, and hence the costs involved; as a result, mortality estimates are rarely available until a year or so after completion of a survey, so for most countries estimates of U5MR are not currently available for recent, short time intervals. This represents a major gap, as annual estimates would be valuable to plan and evaluate the effectiveness of child survival programmes, to target donor funding, and to assess progress against development goals. A faster and less expensive method of generating U5MR estimates would be very useful to researchers and implementers in the health sectors of these countries.

One possible source of more frequent estimates is the Summary Birth History (SBH) which asks each woman surveyed the number of children she has had and the number that have died, along with her age. The simplicity of the questions and of the interviewer training required for a SBH compared with a FBH means that it is cheaper and faster to collect data in this way. In addition, many household surveys not specifically conducted to collect health indicators, such as population censuses, already include SBH data as the necessary questions can be added with little additional cost in terms of interview time or researcher training. There has been a number of estimation methods developed to make use of SBH data in the past [2,3]. However, as SBH estimates are based upon proportions of children dead at the time of a survey, rather than on a full distribution of births and deaths over time, as with an FBH, estimates based on an SBH will not be time- or age- specific since many time paths can lead to the same distribution of proportions dead. Despite this drawback, the potential higher frequency of data collection can be of value.

This paper describes the empirical testing of two methods developed for use in the Rapid Mortality Monitoring (RMM) project of JHSPH [4] which attempt to address this shortcoming of lack of time specificity. An earlier paper [5] describes the methods, and illustrates their application to data collected by or available to the RMM project. In this analysis we apply both methods to new data to assess the performance of the methods under conditions more consistent with the underlying theory.

## Objectives

The two methods based upon SBH, referred to here as the Birth History Imputation method (BHI) and the Cohort Change method (CC), were developed as part of the RMM project. The BHI method imputes a full birth history from another survey onto a summary birth history, matching on children ever born and children dead. The CC method cumulates changes in average numbers of children ever born and average numbers of children dead for cohorts of women between two surveys separated by one or two years. For the RMM project, the methods were tested against the data available in the five project countries, but results were deemed unsatisfactory in terms of the objectives set for the project [5]. However, the project’s study populations, and particularly the data sets to which the methods were applied, were not entirely in line with the requirements of the methods. This paper aims to explore further the performance of the new methods by testing them against the types of data for which they were designed. This is necessary to see if the methods can be rendered usable under suitable conditions and, if found to be invalid, to identify the main sources of error.

This paper is a follow-up to Hill et al [5] published in the RMM supplement to PLOS One. In order to test the performance of these methods, the authors follow three steps:

1. Theoretically test methods using ideal data
2. Real-world tests using best-case-scenario data using survey types most suited to the methods
3. Real-world test in settings with varying quality and suitability of data

The initial testing encompassed steps 1 and 3. In step 1, the methods proved theoretically sound and were immediately applied to the 6 countries in the RMM analysis, in fulfillment of step 3. As described below, these data did not always apply to consistent populations and implementation methods varied. Step 2 was not conducted, and it was therefore unclear whether problems that emerged with the methods, described in Hill et al [5] were a result of the methods themselves, or inconsistencies between the types of data used. By conducting additional analyses upon ‘best-case’ data, we can attempt to answer these questions. All appropriate data sources were considered, including all DHS and MICS applications, and a number of data sources were identified that were most appropriate for the methods.

The BHI method was initially tested using a combination of data sources at different geographic levels for the study country in question. For example in Ethiopia SBH data were collected for the project districts of Jimma and West Hararghe but the FBH data were not available at this level so data for all of Oromia region were used to provide full birth and death distributions. Similar compromises were used for other countries. For this analysis, criteria were that all data sources should relate to national populations, to ensure consistency. In addition, all FBH data used were from DHS and all SBH data were from MICS. This limited the number of countries to those with at least two DHS surveys and one MICS survey carried out between them. We also tested the BHI method for sensitivity to spatial and temporal mismatching, using FBH data sets from a neighboring country (spatial), or from more than 10 years before the SBH survey (temporal), all at national level.

The CC method was initially tested using the data available for the project countries, which included combining DHS, MICS, Census and other survey data, all of which have different implementation methodologies. In several cases, SBH data were generated by summarizing FBH data from a DHS, which may differ significantly from the data as collected by a simple SBH. To test the method fully for this analysis, our criteria were that data should be collected using the same sampling methods and population for each time period, with large sample sizes. We used data from a large, nationally-representative and annually-repeated household survey which used the same survey protocols and methodology repeated over several years from a single country. This generated a series of one- or two-year intervals over which we could compare similar data on a large scale, fitting well with the requirements of this method.

## Methods

### Birth history imputation method

The BHI method requires that an FBH has been collected for the same or similar population at an earlier date. The method imputes a full birth history from the FBH onto each woman in the SBH, controlling for age (or time since first birth if available), children ever born (CEB) and children dead (CD). The procedure is described fully in Hill et al. [5] and has some features similar to methods developed by Montana [6] and Rajaratnam et al [7]. Each mother in the SBH is categorized in terms of the number of CEB, number of CD, and current age, or time since most recent birth. Based on this summary data, a mother who matches on these characteristics is selected at random from the FBH and that mother’s FBH is imputed onto the SBH record. As applied, this procedure is repeated ten times per SBH record in order to reduce random error. Mortality estimates are calculated in terms of years before the survey from the resulting imputed FBH distributions. In the rare case that no match is found in the FBH for a given SBH, the same matching procedure is followed drawing an FBH from a compendium of all DHS FBHs collected across all countries.

The method assumes that the birth intervals and ages at death of children that die in a population change slowly, while the distribution of children ever born and children who have died to women of a given age can change more quickly as mortality changes. The latter change is reflected in the more recent SBH while the former is reflected in the past FBH. Since the imputed FBH is analyzed using standard methods [3], the method can produce age-specific (e.g. neonatal, infant) estimates of mortality, although the confidence intervals around such estimates may be quite large. Estimates appear to be period-specific but are not strictly so, since sudden mortality changes in the period between the FBH and SBH will not be fully reflected; for example, consider an FBH followed one year later by an SBH, and suppose that child mortality was zero in the intervening year; the imputed FBH will still show child deaths in the year before the SBH, because there were child deaths in the year before the FBH; however, the imputed FBH would of course show a decline in child mortality in the year before the SBH, because proportions of dead children would be lower. The method is likely to work best when mortality is changing steadily over time and birth intervals and ages at death are quite stable.

### Cohort change method

The CC method requires data from two SBH surveys at intervals of 1 or 2 years. A relationship can be described between the cumulated cohort changes in average CEB and CD from the first to the second SBH and the Total Fertility Rate and U5MR in the period between the surveys. This method was originally demonstrated by Zlotnik and Hill [8] and modified by Hill et al [5] for one- and two-year periods. Iterative methods for estimating age-specific fertility rates from successive distributions of average CEB by single year of age have been developed by Coale et al. [9] and Stupp [10], and these methods would also be applicable to estimating changes in CD; however, our interest is in total cohort change, not the age pattern of that change. For true cohorts, changes in the number of children ever born from one survey to the next provide a count of all births in the period to the cohort, and changes in number of children dead similarly provide a count of child deaths (at any age) between the surveys. Successive surveys rarely interview the same women, so the method treats cross-sections of maternal age groups as quasi-cohorts, i.e. women aged x in SBH survey year n and women aged x+1 in SBH survey year n+1 are taken to represent a cohort of women born in the year n-x. This method cannot generate age-specific mortality estimates, because the child deaths could occur at any age, but the cohort changes are period-specific. The sum of the changes in CEB across all maternal ages will estimate the period TFR for the interval and the ratio of cumulated change in CD to cumulated change in CEB, CD^c^/CEB^c^, is found empirically to be closely related to the under-5 mortality rate in the period. To avoid distortion due to age-heaping in SBH surveys, rolling 5-year cohorts are used, so for example we group women aged x to x+4 in year n, the corresponding cohort in survey year n+1 being women aged x+1 to x+5; each 5-year cohort therefore includes at least one “attractive” age ending in 0 or 5.

Data from 154 DHS surveys were used to model the relationship between CD^c^/CEB^c^ and the U5MR. A linear relationship was found between the log of the ratio and the log of the U5MR, with adjustment for age distribution of women, the age distribution of child deaths, changes in fertility pattern, and lagged HIV prevalence, which were all found to affect the relationship significantly [5].

### Implementation plan

The BHI method was implemented in 4 countries selected according to the criteria described above (Cameroon, Democratic Republic of Congo (DRC), Ghana and Kenya). For those four countries, the method was applied to 7 national-level SBHs for which appropriate data were available. A full list of these applications can be seen in Table 1. The results reported previously were for 6 applications in 5 countries [5], but only one such application was to national data, and that one application, to Niger, applied the method to an SBH derived from an FBH survey. We chose these country applications as they had UNICEF Multiple Indicator Cluster Survey (MICS) SBH surveys that produced indirect U5MR estimates judged to be of acceptable quality by the internationally recognized UN IGME child mortality monitoring process [11]. They also had DHS FBH surveys available before and after the SBH, as required for imputation and validation.

**Table 1:** Countries and surveys used for estimation applications.

Country	Type of Analysis	Surveys used		
		Summary Birth History	Full Birth History for Imputation	Full Birth History for Validation
Cameroon	Standard test of method	2000 MICS	1998 DHS	2004 DHS
Dem. Rep. Congo	Standard test of method	2010 MICS	2007 DHS	2013 DHS
Ghana	Standard test of method	2006 MICS	2003 DHS	2008 DHS
	Test of contemporaneity	2006 MICS	1993 DHS	2008 DHS
Kenya	Standard test of method	2000 MICS	1998 DHS	2003 DHS
	Standard test of method	2000 MICS	1989 DHS	2003 DHS
	Test of geographic source	2000 MICS	2001 Uganda DHS	2003 DHS

Four of these applications can be regarded as standard, that is, using an FBH from the same national population as the SBH, and for a time period less than 10 years before the SBH. Three applications however are considered non-standard: in two such cases, the FBH used was from a survey conducted more than a decade before the SBH, and in the third case, the FBH came from a survey of a neighboring country (Uganda FBH data used for Kenya SBH imputation).

The CC method was designed to use repeated surveys with the same methodology used over time, and large sample sizes. For the initial RMM study, no such data were available and the failure of the method could have been attributable to inconsistencies between data sources and variable sample sizes. For this study, CC was implemented in one country, Brazil, for which 10 applications were possible, five sets of SBHs at a 1-year interval, and 5 sets of SBHs at a 2-year interval. The source of the data was the Pesquisa Nacional por Amostra de Domicílios (PNAD) from years 2004–2009 and 2011 [12].

The PNAD is a large annual household survey, captures approximately 140,000 households each round, and uses consistent methodologies over time, thus producing data sets ideal for testing the CC method. IGME estimates [11] of under-5 mortality were available for Brazil for the years under consideration, so these were used to validate CC estimates of U5MR.

For Birth History Imputation applications, we estimated U5MR, IMR and NNMR for the 10 calendar years leading up to the year of the SBH. We then compared these individual estimates, as well as the trend over 10 years, with estimates from a best practice existing source, typically FBH estimates calculated using a more recent DHS. Relative errors of individual estimates were computed and as a summary measure we use the mean absolute relative error (MARE). Relative errors were not consistently positive or negative and this diagnostic measure uses absolute values to avoid underestimation of error through positive and negative values cancelling each other out. MARE for each country was calculated as:

$$MARE=\frac{1}{n}\sum_{i=1}^{n}\left|\frac{\left(E_{i}-V_{i}\right)}{V_{i}}\right|$$

where there were n tests of the method, E_i_ is the estimate for test i and V_i_ is the validation value for test i. MARE across all countries was simply the average of country level MAREs.

## Results

The BHI methods resulted in MAREs for U5MR by country ranging from 12.1% to 27.6%, with a median of 15.3%. Aggregating estimates across all countries resulted in an overall MARE of 17.7%. For individual countries, relative errors of estimates above and below validation rates suggested that the mean relative error is approximately zero; however when absolute relative errors are used, the inaccuracy of estimates is clearer. Imputation results can be seen in Table 2. For all estimation methods, an arbitrary standard of ±20% was adopted by the RMM project to determine acceptability of estimates. This allows the BHI method a generous margin of error as both the imputation and validation estimates have sampling errors, so too narrow a band could reject valid estimates. Using this criterion, the number of estimates with relative error within acceptable boundaries ranged from 3/10 in DRC to 9/10 in Kenya. In only two cases are country-level MAREs greater than 20% and only one application, Cameroon, produces a country MARE less than 10%. Pooling all estimates, 43 out of 70 have a relative error within ±20%, so 27 (39%) fall outside the acceptable range. Given such a high failure rate, the accuracy (relative to the validation data set) of the point estimates is not high enough to judge this method to be a success. However there is no clear bias in estimates, with 32 estimates below validation estimates and 38 above.

**Table 2:** Results of birth history imputation applications.

Country	Surveys Used			U5MR Estimates				Number of acceptable estimates
	Summary Birth History	Full Birth History—Imputation	Full Birth History—Verification			Number of Imputed Estimates		
				Mean Relative Error	Mean Absolute Relative Error	20% or more below Verification	20% or more above Verification	
Cameroon	2000 MICS	1998 DHS	2004 DHS	8.8%	15.3%	1	2	7
Dem. Rep. Congo	2010 MICS	2007 DHS	2013 DHS	27.4%	27.6%	0	7	3
Ghana	2006 MICS	2003 DHS	2008 DHS	14.2%	15.2%	0	3	7
	2006 MICS	1993 DHS	2008 DHS	23.7%	23.7%	0	6	4
Kenya	2000 MICS	1998 DHS	2003 DHS	-15.4%	15.4%	3	0	7
	2000 MICS	1989 DHS	2003 DHS	-11.5%	14.7%	4	0	6
	2000 MICS	2001 Uganda DHS	2003 DHS	-11.2%	12.1%	1	0	9

The three applications that do not follow the standard method of using an FBH from the same country from shortly before the SBH survey produced results generally consistent with standard applications. Results from the two applications which used FBHs from a survey more than a decade before the SBH survey are both worse than those using a more contemporaneous FBH. The assumption that birth intervals and ages at death of children do not change in the interval between the FBH and SBH is less likely to hold given such a long lag, so these results are consistent with what we would expect. The application using FBHs from a survey of a neighboring country gives results very similar to the normal application, probably because birth interval distributions and the age distribution of child deaths are similar between Uganda and Kenya, the two countries in question.

These results show no improvement from the previous applications of this method [5]. The previous 5 applications to true SBH data across 4 countries, excluding Niger, resulted in estimates with country MAREs ranging from 10% to 24% and an aggregate MARE of 15%, very similar to the values obtained in this expanded analysis.

As noted above, one of the applications of BHI reported by Hill et al. [5] was to summarized FBH data from Niger, in the format of SBH data. While not a true application of the method, as Niger has not conducted any recent SBH survey, this was a useful test of the method based on a source of data seen as potentially more reliable than a true SBH. The relative errors from the 10 annual estimates of U5MR ranged from 0.1% to 17.7% and the MARE was 7%, substantially better than any applications based on true SBH data. This suggests that the possibly poor, but certainly different, quality of the SBH data may contribute a large proportion of the total error.

To explore this source of error further, comparisons of proportions dead of children ever born by age of mother between the SBH and FBH validation datasets showed large inconsistencies. Where the consistency was better, as with the Malawi application of RMM [5] and to a lesser extent the Cameroon and Kenya MICS 2000, the results were notably better, illustrating that the method can work if CEB and CD data of sufficient quality are available.

For three of the 10 cohort change applications (5 for one-year periods and 5 for 2-year periods), no estimates were possible, as some or all of the maternal age cohorts showed negative cumulated change in children dead. As this is a true cohort method, estimates should be representative of the population and it should be impossible for the average number of children dead of a given cohort of women to decline. However the intervals 2007–08, 2008–09 and 2007–2009 produced negative changes and therefore did not generate valid estimates.

Of the seven estimates possible, two were acceptably close to IGME estimates and five showed large differences [11]. The two acceptable estimates, for periods 2005–06 and 2004–06 gave good results with relative errors of -4% and 6% respectively. Relative errors of the remaining five ranged from -42% to +304%. The full set of results can be seen in Table 3. These results were a slight improvement on the applications reported on previously in which four out of six estimates were impossible and only one was acceptable, with a relative error of 6.4% [5]; note however that none of the previously published results were to consecutive SBH surveys, and therefore could not be regarded as true tests of the method.

**Table 3:** Results of cohort change applications.

PNAD Survey years (Brazil)		Interval (years)	Cumulated Cohort Change		Estimated U5MR ('000)		Relative Error
Start year	End year		CEB	CD	CC	Validation (IGME 2015)	
2004	2005	1	1.95	0.03	13.3	22.8	-42%
2005	2006	1	2.17	0.05	20.4	21.2	-4%
2006	2007	1	2.42	0.24	80.0	19.8	304%
2007	2008	1	1.77	-0.03	a	18.7	a
2008	2009	1	2.06	-0.02	a	17.7	a
2004	2006	2	4.30	0.13	23.2	22.0	6%
2005	2007	2	4.53	0.33	56.8	20.5	177%
2006	2008	2	4.08	0.22	41.5	19.2	116%
2007	2009	2	3.77	0.00	a	18.2	a
2009	2011	2	3.72	0.25	51.4	16.7	208%

^a^. Estimates not possible due to negative changes in children died within some cohorts

## Discussion

We have developed and tested two innovative methods to estimate child mortality for specific time periods based on summary birth histories, using in this paper examples of the most appropriate types of data for each method. This addresses some of the questions raised by the initial tests of the methods detailed in Hill et al [5]. If successful, these methods would allow the rapid estimation of U5MR more cheaply and quickly than current methods, which use large, expensive and infrequent FBH surveys. This would allow for better monitoring and evaluation of child survival programmes, improving targeting of resources and signaling changes to patterns of mortality in the short term. Validation of such methods is scarce in the literature and limited to applications with less than ideal data sets, so this analysis contributes to the field of work.

Unfortunately the results of this analysis show that the methods do not produce acceptably precise estimates with the data available. Of 70 single estimate applications, the BHI method only produced 43 (approximately 60% of estimates) with even a moderately acceptable degree of precision when compared with validation estimates. Even worse, the CC method only produced two acceptable estimates from 10 applications, with three applications impossible to complete. In two cases the estimates were precise, falling within -4% and 6% of IGME validation rates, well within our criterion for acceptability of ±20%.

The use of theoretically appropriate data in these applications rules out some of the potential methodological sources of error which could not be ruled out by previous applications [5]. The source of error for both methods can be primarily attributed to the quality of the SBH data available rather than the methods themselves. Theoretical tests of BHI using Niger FBH data, and of CC using aggregated DHS data, support the validity of these methods; however, such validity was not repeated in country-level applications reported here. The level of consistency between the total proportions of children who had died between SBH and validation surveys was related to the MARE of that application, supporting the conclusion that SBH data quality was a major determinant of error.

Given that the PNAD data should provide a more-or-less perfect application for the cohort change method, the results were a serious disappointment. The method is extremely sensitive to data quality. Even using the PNAD surveys with large samples and SBH data collected using very similar procedures over many years, the SBH data were not consistent between successive surveys, which suggests either poor implementation or insufficient sample size. It is possible that child mortality in Brazil has fallen below a level that can effectively be measured indirectly even by such a large household survey. On the other hand, the fact that both estimates that meet acceptability criteria are very close to validation estimates shows that when data are high quality and consistent, precise estimates can be produced.

This study was not primarily designed as an assessment of SBH data quality. However, an important implication is that users of SBH data should be concerned about data quality. In our analysis, successive SBHs in the same populations with the same theoretical cohorts of women were not consistent with each other. This may be due to recall errors such as omissions or inconsistent reporting of deaths. Studies that aim to assess indirect estimates from SBHs have, arguably, emphasized methods more than data quality. Some studies simulate data without recall errors to assess methodological errors in indirect estimates [13]. Others compare SBH and FBH data from the same sources, such as Silva [14] and Rajaratnam and colleagues [7], using DHS data for both indirect SBH and direct FBH estimates. The probing methods used by DHS data collectors may help to reduce omitted births. Our tests used SBHs from MICS, PNAD, Censuses and one-off surveys that incorporated only SBHs.

This analysis cannot determine how to distinguish a good from a poor quality SBH in the absence of validation data. Standard plausibility checks of female age distribution, sex ratio at birth and parity distribution are recommended, though these checks revealed few serious flaws with DHS SBH data in Silva [14]. To diagnose more precisely the errors arising from implementation of SBH data collection, we would recommend that alternative methods of collecting SBHs be tested. This should be performed at a site with high quality validation data, for example at a Health and Demographic Surveillance System site, and the number of questions, the degree of probing and triangulation as well as the type and extent of training for data collectors could be varied to measure the effects on data quality.

These analyses use a relatively small number of countries and data sources to empirically test the methods. There were a limited number of sources identified that met our criteria, especially for cohort change, and the poor performance of those that were identified did not justify further applications. Should future analysis identify the characteristics of the well-performing SBH data sources that made them successful, or if other data sources that meet the criteria are identified, additional tests may be appropriate.

## Conclusion

In conclusion, summary birth histories do not currently have the potential to produce precise estimates of recent under-5 mortality rates rapidly and cheaply based upon the data used in this analysis. However the negative results of our validation analysis highlight some important lessons. The failure, in most cases, of these methods using ideal data, reveals problems with the quality of SBH data, with implications for other indirect estimation methods that use such data. This conclusion cannot assume the SBH data quality in this analysis necessarily reflects all SBH surveys, though there is nothing to suggest they are atypical. Rather, it calls for research into what makes the consistent SBH applications successful and for additional quality control within SBH data collection. Many household surveys, including population censuses, build in SBH components due to their simplicity; examples are the 2007 population census in Ethiopia [15] and the 2006 MICS Survey in Ghana [16]. If these are so poorly executed as not to be useful, their users and designers should be aware of this when considering the associated costs. In contrast, a few examples of SBH data are shown to be of sufficiently high quality to support these methods, including the Malawi Census 2008 and Cameroon MICS 2000. These practical examples may illustrate ways that SBH data can be improved enough to be useful in future household surveys in LMICs and in the 2020 Census round for rapid child mortality estimation as well as other existing uses. Our theoretical tests of each method based on higher quality data justify their application, should sufficiently high quality SBH data become available in future.
